# Development of reporting guidelines for multiple long-term condition research

**DOI:** 10.1177/26335565261447691

**Published:** 2026-06-12

**Authors:** Hajira Dambha-Miller, Lucy Smith, Clare Macrae, Heather Walker, Glenn Simpson, James Finney, Salwa S. Zghebi, Victoria L. Keevil, Sarah E. Hughes, Rebecca Ross, Kamlesh Khunti, Colin McCowan

**Affiliations:** 1 Primary Care Research Centre, 7423 University of Southampton, Southampton, UK; 2 Centre for Medical Informatics, Usher Institute, University of Edinburgh, Edinburgh, UK; 3 School of Cardiovascular and Metabolic Health, 3526 University of Glasgow, Glasgow, UK; 4 Leicester Diabetes Centre, Leicester General Hospital, 4488 University of Leicester, Leicester, UK; 5 Centre for Primary Care and Health Services Research, 5292 University of Manchester, Manchester, UK; 6 NIHR Exeter Biomedical Research Centre, 3286 University of Exeter, Exeter, UK; 7 Centre for Patient Reported Outcome Research, 1724 University of Birmingham, Birmingham, UK; 8 3129 NHS Lothian, Department of Infectious Diseases, Edinburgh, UK; 9 Leicester Diabetes Centre, 4488 University of Leicester, Leicester, UK; 10 School of Medicine, 7486 University of St Andrews, St Andrews, UK

**Keywords:** multiple long-term conditions, research reporting guidelines, checklist

## Abstract

**Background:**

Incomplete and inconsistent reporting amongst research studies in people with Multiple Long-Term Conditions (MLTC) hinders the comprehensive evaluation, synthesis, and interpretation of study findings for application by clinicians, researchers, patients and policymakers. This limitation leads to heterogeneous findings, duplication of work and restricts the practical application of research outcomes in clinical settings, public health strategies, and policymaking. Given the high prevalence and complexity of MLTC, there is a pressing need for standardised guidelines to promote clarity, consistency, and comprehensiveness in study reporting. Such guidelines can enhance transparency and reproducibility, thereby increasing the impact of research on healthcare decisions and policy development.

**Methods:**

We followed a four-stage process of guidelines development: a review of MLTC reporting practices; a workshop with diverse stakeholders to identify and refine items for inclusion; a prioritisation consensus exercise to agree on key items; and pilot-testing to refine interpretation and usability of the guidelines.

**Results:**

This work has produced the first set of reporting guidelines addressing the need for standardised reporting in MLTC research. Application of these guidelines has the potential to improve research clarity and reproducibility, enabling better comparisons across studies and shared learning. Improved reporting standards will also facilitate the translation of research findings into effective healthcare strategies and policies, contributing to better health outcomes for MLTC patients.

**Conclusion:**

These initial guidelines offer a structured approach to improving the reporting quality of MLTC research. Future evaluations will assess its impact on research transparency and real-world application.

## Background

Multiple Long-Term Conditions (MLTC), defined as the co-occurrence of two or more conditions in an individual, affects a significant and growing portion of the global population.^1^ In the UK, an estimated 19 million (14%) people are affected, with prevalence rates rising as the population ages.^2^ Among those aged over 80 years, 68.2% live with MLTC, leading to complex healthcare needs and significant healthcare management challenges^3,4^ Worldwide, the prevalence of MLTC varies from 16% to 58%, with higher rates observed in older and more vulnerable populations.^5,6^ The increasing prevalence of MLTC contributes to complex care needs, rising costs, and the need for research that integrates multiple disease perspectives rather than focusing on single conditions.^1,7^

This trend has prompted a surge in MLTC research aimed at understanding its complexities and identifying strategies for improved care management. However, limitations in research reporting practices hinder attempts to fully appraise, synthesise, and apply study findings.^8^ Without robust reporting, it is challenging to accurately synthesise findings across studies, limiting the evidence base needed for effective decision-making and care management by patients, healthcare professionals, and policymakers.^9,10^ This lack of transparency and consistency impacts the translation of findings, as critical details about study methodologies, study population characteristics, and outcome measures are frequently underreported or inconsistently presented.^11,12^

A consistent challenge in this field of research is how MLTC is defined and measured. Whilst the standard definition of MLTC is the co-existence of two or more chronic long-term conditions, there remains no consensus regarding which set of conditions should be included in MLTC definitions.^13,14^ Furthermore, there is considerable heterogeneity in the measurement of MLTC, in relation to how conditions should be counted or weighted, which range from simple condition counts to complex indices or clustering approaches.^15^ As Vetrano et al., observe, “being able to adequately operationalize the measurement of multimorbidity in the population represents the basis to fine-tune guidelines”.^16^ Overall, the choice of conditions, the inclusion or exclusion of mental health diagnoses, and the specific threshold used to define MLTC (e.g., ≥2 conditions) can significantly influence prevalence estimates and the interpretation of outcomes.^7^ These variations and inconsistencies constitute significant barriers to comparison across studies, the reproducibility of findings and limit effective meta-analysis and synthesis.

In addition to definitional and measurement inconsistency, the source, quality and variability of data in the current evidence base also present significant reporting challenges.^17,18^ For example, prevalence estimates vary markedly depending on whether data are derived from primary care electronic health records (EHRs), hospital registries, or self-reported surveys.^17–19^ These data sources differ in completeness, coding precision, and the ability to capture the temporality and severity of disease.^20^ Without transparent reporting of data provenance, it becomes difficult to interpret the comparability and generalisability of MLTC studies.^20,21^

Recognising these limitations, the need for standardised MLTC reporting guidelines has become increasingly urgent. Transparent and comprehensive reporting frameworks can enhance consistency, reproducibility, facilitate more reliable evidence synthesis, accelerate the pace of research and ensure research outcomes are relevant for real-world applications.^22,23^ Organisations such as the EQUATOR Network, which advocates for quality in health research, have successfully introduced guidelines in other research areas, including CONSORT for clinical trials and STROBE for observational studies.^24,25^ In the context of MLTC, where study complexity and diverse patient populations create additional challenges, reporting guidelines specific to this population, alongside existing methodological standards, may help establish consistency. This would enable stakeholders to better understand and interpret findings, enhancing the impact of MLTC research on healthcare policy and practice.^26^

In this study, we aimed to identify and address reporting gaps by developing a structured reporting guideline specifically for MLTC research. Such a guideline would provide researchers with a clear framework for reporting essential study details, improving transparency, reproducibility, and the potential of research findings to inform clinical practice more effectively.

## Methods

**Figure 1.**
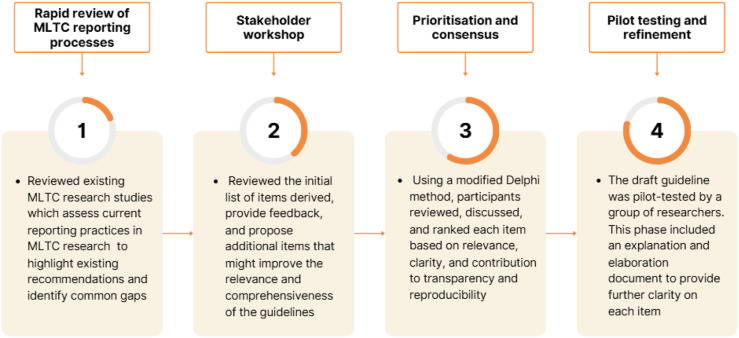
Flow chart showing the four-stage process for guideline development on transparent reporting of MLTC research.

To develop this guideline on transparent MLTC research reporting, an established four-stage process for guideline development was adopted.^22^ This included a rapid review, stakeholder workshop, prioritisation consensus exercise, and pilot testing to ensure the guideline’s clarity, relevance, and usability (see Figure 1). Whilst this model is linear, the rapid review process was conducted in parallel with stages two and three, helping to inform the stage four output.

### Stage 1: Rapid review of MLTC reporting practices

Stage one involved a rapid review of existing MLTC research to assess current reporting practices, identify existing recommendations and potential gaps in reporting.^27,28^ A rapid review is a “form of knowledge synthesis that follows a streamlined process to produce evidence summaries in a timely manner … [and] are designed to be more agile and responsive and completed in less time compared to conventional, full systematic reviews”.^27^ The scope of rapid reviews is often narrow, focused on specific research questions or populations, and they use methods designed to ensure timely delivery of review objectives.^28^

Search terms included MeSH terms related to MLTC and reporting guidelines (see Appendix One). Comprehensive searches were conducted on PubMed, EMBASE, CINAHL and Web of Science, covering literature from inception to May 27^th^, 2025. Searches were conducted by SZ, CM, HW, SH and RR and LS and transferred to the Rayyan platform. Duplicates were removed and abstracts screened by LS and HW. Full texts of papers were screened by LS and HW and disagreement about including papers was arbitrated by a third reviewer, CM. In addition to database searches, CM and RR examined grey literature sources, using Google Scholar, hand-searched key journals and reference lists and consulted with subject experts.

Data extraction focused on identifying recurring issues in reporting, including suggestions about the reporting of participant characteristics, operationalisation of chronic conditions, outcome measures, study designs, and/or intervention descriptions. The findings informed the initial list of candidate items for potential inclusion in the reporting guideline (see Appendix Two).

### Stage 2: Stakeholder workshop

In stage two, a two-day workshop was held in early 2024, with 23 stakeholders from across the UK, with backgrounds and expertise in data science, epidemiology, clinical trials management, quality improvement, social sciences, healthcare management, clinical practice, industry, patient advocacy groups, policymakers, patients, and carers of people with MLTC. Stakeholders were invited to the workshop based on their subject-matter expertise, experience in conducting MLTC research, understanding of national and local policy, and professional roles within MLTC-related health services. Participants represented a broad range of sectors and professional backgrounds, offering local, regional, and national perspectives. The group included seven care professionals and public health practitioners, two programme managers, and two epidemiologists, as well as one representative each from industry, policy, health economics, methodology, and theoretical physics. In addition, two individuals with lived experience of MLTC and/or caring responsibilities were involved, recruited through an open call to ensure inclusion of both patient and informal carer perspectives.

The workshop was facilitated by four specialists in participatory research methods, two of whom also had specific expertise in MLTC. Following the workshop, the collected data were transcribed and analysed thematically. Researchers first conducted an open coding process to identify recurring ideas and patterns in the data, followed by axial coding to group related themes. Further analysis refined the themes into higher-order categories. Three researchers independently reviewed the codes to ensure consistency and reliability throughout the analysis.

The purpose of the workshop was to review the initial list of items derived, provide feedback, and propose additional items to improve the relevance and comprehensiveness of the guidelines. Stakeholders evaluated each item for clarity, applicability, and importance, focusing on aspects critical to improving reporting quality in MLTC research. This step ensured the guidelines would reflect a wide range of perspectives and address real-world challenges in MLTC research reporting.

### Stage 3: Prioritisation and consensus

The third stage involved a structured consensus process to finalise the key items for inclusion in the reporting guidelines. This included an initial in-person consensus meeting, followed by an online meeting, both involving a multidisciplinary panel of stakeholders, including clinical researchers, methodologists, healthcare professionals, policy experts, and individuals with MLTC and their caregivers.

A modified Delphi method was used to systematically prioritise items identified during earlier stages of the project.^29^ Participants reviewed, discussed, and independently rated each item based on relevance to MLTC research, potential to enhance transparency, reproducibility, and methodological rigour. The prioritisation exercise involved four rounds of voting. After each round, results were presented to the group to facilitate discussion, clarification, and refinement of items. Items were re-evaluated in subsequent rounds to work toward consensus. Consensus was defined as ≥70% agreement on the inclusion or exclusion of an item. This iterative, feedback-driven process enabled convergence of expert opinion, ensuring that the final set of items reflected both empirical evidence and the collective judgement of a diverse panel.

### Stage 4: Pilot testing and refinement

In the final stage, the draft guideline was piloted to ensure it could be operationalised in MLTC research. Feedback was collected on its ease of use, clarity of instructions, and completeness of information required for effective reporting. Based on the feedback, minor revisions were made to improve the guideline’s usability and interpretation. This iterative refinement ensured that the final guideline was practical for MLTC research and effectively promoted more consistent and transparent reporting.

## Results

### Stage 1: Summary of rapid review findings

**Figure 2.**
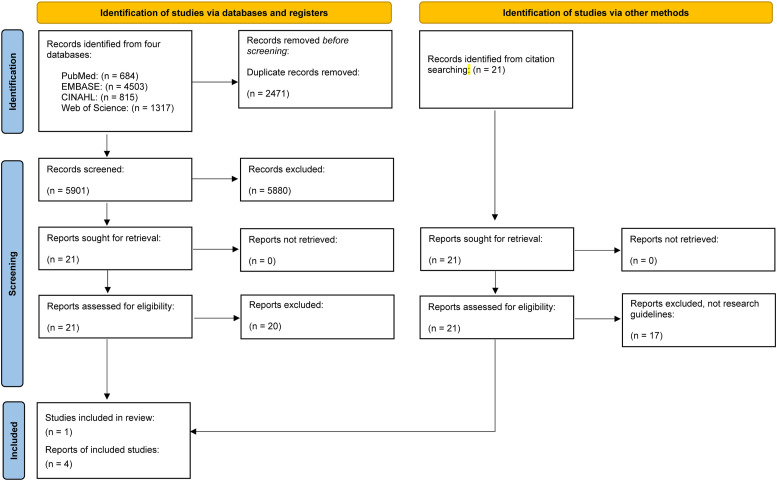
Prisma flow diagram.

A total of 7,304 records were identified. After removing duplicates and screening the titles, abstracts, and full texts, five papers met the inclusion criteria and were included in the final synthesis (Figure 2).

Overall, the evidence on reporting practices in MLTC research was limited. No included studies provided explicit recommendations regarding the reporting of participant characteristics or the reporting of non-randomised or randomised study designs. One study^30^ used a Delphi study methodology to provide guidance on important outcome measures for MLTC studies, recommending quality of life, mental health outcomes, and mortality, while noting that outcome selection should be context-dependent and aligned with study objectives. Three studies^13,31,32^ examined the classification and inclusion of long-term conditions, proposing varying thresholds: one identified 60 conditions, another 59, and the third 32. A Delphi study conducted by Wang et al. identified 24 core long-term conditions recommended for inclusion in MLTC measures, alongside a further 35 long-term conditions that should generally be included unless a clear justification for exclusion exists.^33^ Reporting guidance related to outcome measures and intervention descriptions was scarce. One study, by Calderon-Larranga et al., aimed to comprehensively address the operationalisation of MLTC by recommending steps that researchers should take to enhance transparency of research.^31^ The authors recommend that conditions included within counts of MLTC must meet their definition of chronic (or long-term) which was if the condition “had a prolonged duration and either (a) left residual disability or worsening quality of life or (b) required a long period of care, treatment, or rehabilitation”. Based on this definition, they provide a list of 60 long-term conditions with accompanying ICD-10 code lists for use by researchers when measuring MLTCs in research. This guideline is a major step forward for improving the consistency of MLTC research. However, the study recommends reporting methods that are only for use in research that examines MLTC in older adult populations aged 60 years and over, and provides code-lists that can be applied to hospital inpatient electronic health records and prescribing data according to Anatomical Therapeutic Chemical (ATC) categories without inclusion of primary care or self-report datasets. One study by Johnston et al.^6^ recommends that researchers use the most applied definition of two or more long-term conditions, however, were general in their recommendation for the choice of conditions such that researchers should use condition lists that have been validated according to the study outcome.

In conclusion, the existing literature is limited and does not provide recommendations for the pipeline of the development of MLTC research from conceptualisation to implementation across a wide range of research study settings and data sources, and although outcomes appropriate to MLTC research are well-described it is less clear which conditions to include when studying each outcome. Our study responds to these gaps by recommending consistent approaches to reporting demographic characteristics of study populations, methods used when analysing data ranging from presenting code-lists used for the constituent conditions, measurement of study outcomes, and inclusion of PPI stakeholders with experience of MLTCs.

### Stage 2: Stakeholder workshop findings

The workshop had three key objectives: to identify methodological challenges in MLTC research; to prioritise critical areas that require improvement; and to consider strategies to strengthen research methodologies in this field. Several key recommendations emerged from the workshop. First, a call for standardised outcome measures for MLTC research, with an emphasis on aligning outcome measures with patient-prioritised metrics. Participants recommended more nuanced classification systems that account for disease severity and improve both transparency and reproducibility in MLTC research practices. The establishment of a centralised MLTC data repository was proposed to support collaboration and enhance research efficiency. Additionally, participants noted a significant gap in training and mentorship opportunities, calling for more capacity in this area. Strengthening collaboration between academic and non-academic sectors was considered essential for advancing the field. Overall, there was strong support for developing dedicated reporting guidelines for MLTC research, similar to the EQUATOR guidelines.

### Stage 3: Draft guidelines and revisions

The guidelines were developed with detailed recommendations across five primary domains: (1) Defining MLTC, (2) Population Profile, (3) Methodology, (4) Outcome, and (5) Patient and Public Involvement and Engagement (PPIE).

### Domain 1. Defining MLTC

Researchers should clearly define what constitutes MLTC or the related term they decide to use in their study and explain how it is operationalised. Terminology must be applied consistently throughout the study. This is essential to ensure consistent and comparable results across studies. Researchers should address the following core components:

#### Long-term condition inclusion

• Provide a comprehensive list of all conditions included in the study’s definition of MLTC and state whether the NIHR-adopted 59 ‘must-have’ core conditions list was used. If not feasible due to data source or study design constraints, clearly justify deviations, including which conditions were excluded and the implications for interpretation and comparability.
• Clearly describe how these conditions were operationalised to measure MLTC (e.g., whether they were counted, weighted, or grouped).
• If using a pre-existing condition list to support the inclusion of certain conditions, the source of this list should be explicitly stated. If modifications, deviations, or adaptations were made to any existing list (e.g., removing certain conditions, adding others, or modifying thresholds), these must be clearly described.
• Each included condition should be defined using a consistent and reliable source. For example, researchers may use standardised coding systems such as ICD codes, SNOMED CT, or self-reported data. For self-reported data, outline the methodology clearly. The source of condition identification must be explicitly stated.
• If conditions are grouped (i.e.,‘lumping’) or separated into finer categories (i.e.,‘splitting’), researchers should provide a transparent explanation of the criteria used for these decisions. For example, if multiple related conditions are grouped as a single entity, the rationale should be explained (e.g., clinical relevance or patient perspectives). Similarly, if a single condition is split into subgroups, researchers should describe how these divisions are made (e.g., based on severity, type, or other factors).

#### MLTC status

• Define the specific threshold used to identify MLTC in the study (e.g., the number of conditions required to meet the definition). Ensuring that Researchers’ definition of a ‘long-term condition’ or ‘chronic condition’, etc, is also reported (e.g. a condition present, or expected to be present, for at least 12 months). Researchers should explain the chosen threshold and why it was selected based on the study’s objectives and focus.
• Indicate if and which mental health and/or physical health conditions are included in the definition of MLTC. Researchers should state whether mental health conditions (e.g., depression, anxiety, schizophrenia) are included (and provide justification) or any other conditions that are typically excluded from the MLTC count (e.g., acute infections, conditions considered short-term/non-chronic, etc).
• If specific conditions are excluded, justify these exclusions. For example, some studies might exclude specific conditions because they have a distinct pathophysiological course, are not typically considered chronic, or are treated separately in the context of MLTC studies.

##### Temporality/Duration and severity

• Report the duration of each condition and consider temporality when defining MLTC. Specify the time period during which conditions must be present to be counted as part of MLTC status (e.g., diagnosed within the past five years). Also, discuss whether a ‘look-back’ period was used to account for conditions that have been actively managed within a set period. Address the sequence of diagnoses, especially if the order in which conditions were diagnosed could influence the understanding of their interrelationships. Clarify how temporality is considered, particularly in studies exploring interactions over time.
• Indicate whether condition severity was considered and clearly describe how severity was defined or measured (e.g., symptom severity, functional impairment, clinical guidance, or end-stage complication).
• In studies on the interaction of multiple conditions, researchers should explain how the severity of each condition influences the overall definition of MLTC, including whether severity is factored into the analysis.

##### Definition consistency

• Ensure the terminology used in defining MLTC remains consistent throughout the study. Fluctuating terms such as “comorbidities,” “long-term conditions,” or “multimorbidity” can introduce confusion and reduce comparability of results across studies. Additionally, the term multimorbidity has received considerable negativity from patient groups, as morbidity implies fatality.^34^ Provide a clear and consistent definition from the outset of the study and apply it throughout the research process.
• The term MLTC must be defined in a way that reflects the study’s approach, whether defined purely by the number of conditions, the nature of the conditions, or other factors such as severity or duration.
• Researchers should clearly explain how definitions are applied and justify any deviations from conventional terminology. Where necessary, researchers are encouraged to provide examples to clarify their approach, to ensure definitions are clear and understandable to both the research community and other stakeholders.

### Domain 2. Population profile

Comprehensive reporting of the patient cohort characteristics is critical for assessing the representativeness and relevance of the study sample. Researchers should ensure transparency in describing how the sample was selected and provide detailed demographic and socio-economic information. The following elements should be reported:

#### Data source

• If the study involves secondary data analysis, researchers should clearly describe the data sources, such as routinely collected health or social care data, and the method of access. For example, if the data is drawn from health records, national registries, or insurance databases, provide these details.^35^
• If primary data collection methods were used, outline the recruitment process, including how participants were selected, the inclusion and exclusion criteria, and whether informed consent was obtained. This helps clarify whether the study sample is representative of the general MLTC population.

#### Demographics

• Report the mean age of participants with age categories (e.g., 18-30, 31-45, 46-60, 61-75, 76-90, >90) and compare the study sample to the broader MLTC population in terms of age distribution.
• Report sex, indicating whether the data are self-reported or based on biological sex.
• Depending on the scope of the work, provide and report ethnicity data in alignment with relevant national or international guidance and other widely accepted guidelines. If ethnicity data is missing, researchers should acknowledge the extent of any missing data and describe how it may affect the study’s findings. The analysis should consider the implications of non-representative ethnic samples.
• Discuss how representative the sample is of broader MLTC demographics, noting key gaps or biases, including protected characteristics.

#### Socioeconomic status

• Report socioeconomic characteristics to provide context for the study population’s social determinants of health. Key variables include employment status (e.g., employed, unemployed, retired) and measures of deprivation (e.g., Index of Multiple Deprivation or Townsend scores).
• Specify whether deprivation measures were used and discuss the implications for interpretation of the findings.

#### Geographic location

• Report the geographical location of participants in detail, including urban vs rural distinctions, regions, or specific countries.
• Where possible, the sample’s geographic diversity should be considered when interpreting the results, particularly if the study focuses on specific regional or national trends in MLTC.
• Consider assessing whether the findings are generalisable to different geographical settings or populations.

#### Diagnosis timing

• Where possible, report the timing of participants’ diagnoses, particularly when they were first diagnosed with MLTC. Linking diagnosis timing to cohort characteristics provides context to the study, as the timing of diagnosis can impact treatment pathways and outcomes.

### Domain 3. Methodology

This domain guides conducting research in a transparent, replicable, and methodologically sound manner, specifically in relation to MLTC analytical approaches and code transparency**.**

#### Analytical methods for condition combinations

• Provide detailed information on the analytical techniques used to identify or combine conditions.
• If clustering methods or AI algorithms are employed (e.g., hierarchical clustering, k-means, machine learning algorithms like random forests or support vector machines), these should be described, including the rationale for choosing specific methods.
• If clinical expertise or patient-derived data are used to determine combinations, this should be explicitly stated, along with an explanation of the process (e.g., expert/Delphi panels, patient interviews).
• State key assumptions underlying the analytical methods and discuss how these may influence interpretation and generalisability.

#### Code lists and validation

• Researchers must clarify the source of any disease codes used in the study. If existing coding systems (e.g., ICD, SNOMED) are used, the exact version or update date should be specified.
• If novel codes are introduced, the rationale for their development and any associated validation processes must be described.
• If practicable, the validation process may involve expert clinical review, cross-validation with other data sources, or direct input from Patient and Public Involvement and Engagement groups to ensure the codes accurately reflect the conditions being studied.
• Justify deviations from standard code lists with reference to clinical or methodological considerations.

#### Reusability and analytical code

• To ensure that research is transparent and reproducible, make the analytical code publicly available, ideally in an open-access repository such as GitHub, where others can access, use, and build upon it.
• The code should be accompanied by clear documentation on how to use it and any dependencies required (e.g., software versions, libraries).
• State whether the code is open-source and outline any licensing conditions for reuse.

### Domain 4. Outcomes

This section provides guidance on selecting, reporting, and justifying the outcome measures used in MLTC studies.

#### Core outcome dataset usage

• Indicate whether the MLTC Core Outcome Set was used.
• Specify which core outcome set was used and its current validity and appropriateness for the study population.
• If no MLTC Core Outcome Set was used, provide a clear rationale for deviation.

#### Outcome relevance

• Justify the selection of each outcome in relation to the study’s primary research question.
• For each outcome measure, describe whether it is subjective, objective or validated.
• Clarify the methodology used to collect data on each outcome.

#### Stakeholder relevance

• Specify which stakeholder groups (e.g., patients, carers, healthcare providers, policymakers) each outcome is most relevant to.
• Where possible, ensure the outcomes are inclusive and consider the potential for bias.

#### Equality, diversity, and inclusion (EDI)

• Discuss potential biases and equity, diversity, and inclusivity considerations associated with outcome measurement in MLTC populations.
• Where possible, provide a rationale for how EDI considerations were incorporated into the outcome selection process.

### Domain 5. Patient and public involvement and engagement (PPIE)

#### PPIE reporting

• Describe whether and how patients living with MLTC and members of the public were involved throughout the study (design, analysis and dissemination).
• Explain the impact of their involvement on study decisions, interpretation, or dissemination.
• In relation to PPIE, document knowledge exchange workshops and explain how wider engagement, post-study, has influenced future knowledge mobilisation and implementation strategy.
• Have a clear strategy for how equity, diversity, and inclusion are considered within all PPIE practices, to ensure diverse perspectives are represented and engagement is inclusive of all relevant groups.^36^

### Stage 4: Final guideline

Based on the developmental work conducted, we developed the formal consensus-based reporting statement (Table 1).

**Table 1.** Reporting statement: Core checklist of items that should be included in reporting multiple long-term conditions (MLTC) research.

​	Item no	Core reporting recommendations
Defining MLTC	**1**	(a) Specify how the term multiple long-term conditions (MLTC) or a related term (e.g., multiple chronic conditions) is defined in the study, including specifying the physical and/or mental conditions considered and the rationale for inclusion. Apply the term consistently throughout the study.
		(b) State whether the NIHR-adopted 59 ‘must-have’ core conditions list was used as a reference framework. This represents a consensus-informed standard within the UK context and is not intended as a universal or definitive gold standard. Where alternative condition lists are used, clearly justify their selection and discuss implications for comparability.
		(c) Describe how conditions were identified and the source, including specifying any coding or classification systems used (e.g., self-reported, ICD, SNOMED or clinical guidelines).
		(d) Indicate whether the severity of conditions was considered, and how this was determined (e.g., clinical guidance, symptoms or end-stage complication).
		(e) Describe how the temporality/duration of conditions (e.g., look-back periods or order of onset) were considered.
Population profile of MLTC population	**2**	(a) Describe key demographic characteristics relevant to MLTC interpretation, including age, sex (indicating whether the data are self-reported or based on biological sex) and ethnicity (with missingness), and their implications for equity.
		(b) Specify whether deprivation measures were used (e.g., IMD, Townsend Score), including implications for interpretation of the findings.
		(c) Where possible, report geographic information and context (e.g., region, urban/rural classification) of the study population.
		(d) If other population characteristics are considered (e.g., occupation, residential status), provide a rationale for their inclusion.
Methods specific to MLTC	**3**	(a) Ensure that the analytical methods used to inform combinations of conditions in the MLTC population have been clearly specified (e.g., which clustering type or AI algorithm is used), including key assumptions. If disease combinations are determined by other means, these should be reported, and the rationale explained (e.g., clinical opinion, patient-derived).
		(b) If disease code lists were used, report their source and any clinical validation undertaken, or if novel, explain any deviation from existing code lists.
		(c) Indicate whether analytical code or MLTC code lists are open source and publicly available (e.g., deposited in GitHub).
Outcomes	**4**	(a) Specify if the MLTC Core Outcome set has been used or provide a rationale for any deviation.
		(b) Indicate which MLTC stakeholder groups (e.g., patients, carers, clinicians or policymakers) each outcome is most relevant to.
		(c) Discuss potential biases and equity, diversity, and inclusivity considerations associated with outcome measurement in MLTC populations.
Patient and public inclusion	**5**	Describe whether and how patients living with MLTC and the public were involved throughout the study (e.g., design, analysis and dissemination), and explain the impact of their involvement. If absent, explain the justification for non-involvement.

## Discussion

To our knowledge, these are the first comprehensive guidelines addressing the need for standardised reporting in MLTC research. The growing burden of MLTC, particularly among older populations, demonstrates the need for high-quality, transparent research which can inform clinical practice and policy. As the global prevalence of MLTC continues to rise, so does the volume of research aimed at understanding and managing these conditions. This explosion of MLTC research makes it increasingly difficult to synthesise findings, making clear, consistent reporting essential.

The findings from our work highlight the importance of reporting both how long-term conditions were defined and sourced (e.g., the clinical consensus, prior frameworks, ICD/SNOMED code groups, etc), and how MLTC was measured and operationalised in line with a study’s specific rationale and objectives (e.g., simple counts and thresholds, complex weighted indices, data-driven clusters or accumulation rates, etc.). Clear separation of these steps enables transparency and provides conceptual clarity in MLTC research, improving comparability and synthesis across studies.

Overall, focusing on key areas identified through our consensus-based approach, these guidelines will enhance the overall quality and utility of MLTC research. Furthermore, from care and service delivery perspectives, by improving reporting standards in MLTC research, these guidelines also facilitate the translation of research findings into effective healthcare strategies and policies to better address the growing care needs of MLTC patients. These proposed reporting guidelines are designed to complement, rather than replace, existing frameworks such as STROBE, CONSORT, RECORD, and PRISMA.

## Comparison to existing guidelines

The MLTC Reporting Guidelines presented in this work complement established reporting frameworks such as STROBE^37^ and CONSORT,^38^ while filling a gap by addressing the specific complexities inherent to MLTC research. These existing guidelines are predominantly designed for studies focusing on single conditions or conventional clinical trials and fail to capture the methodological and conceptual challenges unique to MLTC, where multiple, interacting conditions must be accounted for simultaneously. High-quality evidence from trials remains essential for improving the management of MLTC, although such evidence is currently limited.^39^ Among the five studies included in our review, each offered recommendations addressing specific aspects of research design or methodology. While these contributions were valuable, they tended to focus on isolated components rather than providing an integrated approach to the reporting needs of MLTC research. Our current guidelines fill this gap by providing more comprehensive, tailored recommendations that address key underrepresented areas. These include clearer definitions of MLTC, attention to disease severity and temporality, the systematic reporting of diverse patient demographics and consideration of how MLTC is operationalised, elements that are frequently overlooked or inconsistently reported in existing literature.^40^ The guidelines also emphasise the inclusion of PPIE throughout the research lifecycle and the use of core outcome sets. These features are essential for ensuring research remains aligned with the needs and values of patients and other stakeholders. Importantly, none of the earlier work identified in our rapid review addressed the representation of diverse populations or the integration of PPIE, both of which are essential for promoting health equity and the relevance and utility of research findings.^41^ The absence of such considerations can hinder knowledge mobilisation and perpetuate health disparities, as broader societal and contextual factors are often neglected in the design and reporting of MLTC studies.^40^ By incorporating these critical dimensions, our work offers a more holistic and context-sensitive approach to reporting in MLTC research. These guidelines will support transparency, inclusivity, and real-world applicability in a field where complexity is the norm rather than the exception.

## Strengths and limitations

This study is the first to present comprehensive reporting guidelines for MLTC research. The strengths of the MLTC Reporting Guidelines are their methodological rigour and involvement of a diverse range of expert input that informed their development. The use of a rapid review, stakeholder consultations, and a consensus-building exercise ensured that the guidelines are both comprehensive and grounded in real-world practice. This inclusive process also ensured that the guidelines are relevant across various contexts and research settings.

There are some limitations. As with all consensus-based research, the findings are influenced by the composition of the sample, which in this case was limited to contributors from the UK. This sample lacked representation from low- and middle-income countries, although some panel members had experience of research in these regions. Additionally, the inclusion of diverse stakeholders may have prioritised certain perspectives over others, and our workshop may not have captured the full range of perspectives due to the relatively limited sample size. For example, frontline General Practitioners and other allied professionals were fewer than intended due to clinical workloads, and participation from local government social care, third-sector providers, and commissioners varied by region. Informal carer and patient perspectives were included but were limited in number. Therefore, future work should assess wider applicability, especially across different countries and whether the identified elements lead to greater transparency and robustness of future work. The stakeholder group was primarily UK-based, reflecting the funding and policy context within which this reporting guideline was developed. We recognise that broader international representation will be important in future phases to support validation and applicability across different healthcare systems and research settings. Additionally, the guidelines may face challenges when applied to studies conducted in different healthcare systems, where contextual adaptations may be necessary. Restricting our research to studies using only the English language may have led to other important work written in other languages being excluded from the development of these reporting guidelines.

## Conclusion and future work

Further testing of the guidelines is essential to assess their real-world impact on MLTC research. This could involve conducting studies using these guidelines to examine whether they improve the quality, comparability, and reproducibility of MLTC research outcomes. Feedback from researchers, clinicians, and policymakers should be used to revise and refine the guidelines periodically, ensuring they remain up to date with emerging research and evolving healthcare needs. Establishing links with international organisations, such as the EQUATOR Network, will be crucial for further developing and promoting the global adoption of these guidelines. Regular updates and revisions based on ongoing feedback will strengthen their applicability, ensuring that MLTC research continues to inform clinical practice and policy in a meaningful way.

## Data Availability

All data used for this research will be made available upon reasonable request to the authors.*

## Appendix

### Appendix one: MeSH terms table

**Table:**

MeSH and search terms	Databases searched	Filters	Number of returns
“multimorbidit*” or “multi-morbidit*” or “comorbidit*” or “co-morbidit*” or “polymorbidit*” or “poly-morbidit*” or “multicondition*” or “multi-condition*” or “multiple chronic condition*” or “morbidity burden*” or “multiple long-term condition*” or ((multiple or coexisting or co-existing or concurrent or con-current or comorbid* or co-morbid* or several*) adj2 (disease* or illness* or condition* or diagnosis* or morbid* or patholog*))).mp. AND guideline* or method* or “research design” or “reporting guideline*” or “reporting standard*” or framework* or “consensus guideline*”) adj3 (report* or “good practice”)).tw.	EMBASE	English language only	4503
TI (“multimorbidit*” OR “multi-morbidit*” OR “comorbidit*” OR “co-morbidit*” OR “polymorbidit*” OR “poly-morbidit*” OR “multicondition*” OR “multi-condition*” OR “multiple chronic condition*” OR “morbidity burden*” OR “multiple long-term condition*” OR ((multiple OR coexisting OR co-existing OR concurrent OR con-current OR comorbid* OR co-morbid* OR several*) N2 (disease* OR illness* OR condition* OR diagnosis* OR morbid* OR patholog*))))	CINAHL (EBSCO interface)	English language only	815
AB (“multimorbidit*” OR “multi-morbidit*” OR “comorbidit*” OR “co-morbidit*” OR “polymorbidit*” OR “poly-morbidit*” OR “multicondition*” OR “multi-condition*” OR “multiple chronic condition*” OR “morbidity burden*” OR “multiple long-term condition*” OR ((multiple OR coexisting OR co-existing OR concurrent OR con-current OR comorbid* OR co-morbid* OR several*) N2 (disease* OR illness* OR condition* OR diagnosis* OR morbid* OR patholog*)))) SU (“multimorbidit*” OR “multi-morbidit*” OR “comorbidit*” OR “co-morbidit*” OR “polymorbidit*” OR “poly-morbidit*” OR “multicondition*” OR “multi-condition*” OR “multiple chronic condition*” OR “morbidity burden*” OR “multiple long-term condition*” OR ((multiple OR coexisting OR co-existing OR concurrent OR con-current OR comorbid* OR co-morbid* OR several*) N2 (disease* OR illness* OR condition* OR diagnosis* OR morbid* OR patholog*)))) TI (guideline* OR method* OR “research design” OR “reporting guideline*” OR “reporting standard*” OR framework* OR “consensus guideline*”) N3 (report* or “good practice”))) AB (guideline* OR method* OR “research design” OR “reporting guideline*” OR “reporting standard*” OR framework* OR “consensus guideline*”) N3 (report* or “good practice”))) SU (guideline* OR method* OR “research design” OR “reporting guideline*” OR “reporting standard*” OR framework* OR “consensus guideline*”) N3 (report* or “good practice”))) S5 OR S6 OR S7 S4 AND S8	Web of Science and National Institute for Health and Care Excellence	​	1317
((“multimorbidity” [MeSH Terms] OR “comorbidity” [MeSH Terms] OR “multimorbidity” [TIAB] OR “multi-morbidity” [TIAB] OR “comorbidity” [TIAB] OR “co-morbidity” [TIAB] OR “polymorbidity” [TIAB] OR “poly-morbidity” [TIAB] OR “multicondition” [TIAB] OR “multi-condition” [TIAB] OR “multiple chronic condition” [TIAB] OR “morbidity burden” [TIAB] OR “multiple long-term condition” [TIAB] OR ((multiple OR coexisting OR “co-existing” OR concurrent OR “con-current” OR comorbid OR “co-morbid” OR several) AND (disease OR illness OR condition OR diagnosis OR morbid OR pathology))) AND (((“Practice Guidelines as Topic” [MeSH Terms] OR “Reporting Standards” [MeSH Terms] OR “Consensus Development Conference” [MeSH Terms] OR “Consensus Guidelines” [MeSH Terms]) OR (“reporting guideline” [TIAB] OR “reporting standard” [TIAB] OR “consensus guideline” [TIAB])) AND (“report” OR “good practice”)))	PUBMED	English language only	684
Google Scholar	Hand searches	English language only	21
**Total number of papers**	​	​	**7304**

### Appendix two: Extraction table for rapid review

**Table:**

Title and author	Year	Reporting participant characteristics	Operationalisation of chronic conditions/multimorbidity	Reporting study design	Reporting outcome measures	Reporting trial design	Reporting intervention design
Assessing and Measuring Chronic Multimorbidity in the Older Population: A Proposal for Its Operationalization Calderon-Larranga et al.,	2017	n/a	“A disease or condition was considered to be chronic if it had a prolonged duration and either (a) left residual disability or worsening quality of life or (b) required a long period of care, treatment, or rehabilitation. After applying this definition in relation to populations of older adults, 918 chronic ICD-10 codes were identified and grouped into 60 chronic disease categories. In SNAC-K, 88.6% had ≥2 of these 60 disease categories, 73.2% had ≥3, and 55.8% had ≥4.”	n/a	n/a	n/a	n/a
Adapting the definition of multimorbidity – development of a locality-based consensus for selecting included Long Term Conditions Hafezparast et al.,	2023	n/a	To define multimorbidity for an inner city, multi-ethnic, deprived, young age community typical of many large cities, 32 LTCs were suggested.	n/a	n/a	n/a	n/a
Measuring multimorbidity in research: Delphi consensus study Ho et al.,	2022	n/a	The consensus was that multimorbidity should be defined as two or more long-term conditions. “conditions should be included in a multimorbidity measure if they were one or more of the following: currently active; permanent in their effects; requiring current treatment, care, or therapy; requiring surveillance; or relapsing-remitting conditions requiring ongoing care. Consensus was reached for 24 conditions to always include in multimorbidity measures, and 35 conditions to usually include unless a good reason not to existed. Simple counts were preferred for estimating prevalence and examining clustering or trajectories, and weighted measures were preferred for risk adjustment and outcome prediction.”	n/a	n/a	n/a	n/a
Strengthening transparency in randomised trials related to multimorbidity: key points and recommendations to guide reporting Wang et al.,	2024	n/a	n/a	n/a	n/a	“4 key points raised: 1. The multimorbidity aspect should be clearly identifiable. 2. It should be specified whether the interventions aim to target a combination of conditions or only certain condition(s). 3. The definition of multimorbidity and the related eligibility criteria should be declared precisely. 4. The baseline characteristics related to the different comorbidities should be reported. 5. The interaction between the interventions, different morbidities and the general condition of the patient needs to be considered, and possible benefits should be balanced with harms considering all comorbidities.”	n/a
A Core Outcome Set for Multimorbidity Research (COSmm). Smith et al.,	2018	n/a	n/a	n/a	“The Delphi panel reached consensus on 17 outcomes for inclusion in a core outcome set for multimorbidity (COSmm). The highest-ranked outcomes were health-related quality of life, mental health outcomes, and mortality. Other outcomes were grouped into overarching themes of patient-reported impacts and behaviors (treatment burden, self-rated health, self-management behavior, self-efficacy, adherence); physical activity and function (activities of daily living, physical function, physical activity); consultation related (communication, shared decision making, prioritization); and health systems (health care use, costs, quality of health care).”	n/a	n/a
